# An Account of the Last Illness of the Late Honorable Daniel Webster, Secretary of State: With a Description of the Post-mortem Appearances, &c.

**Published:** 1853-03

**Authors:** John Jeffries


*An Account of the last Illness of the late Honorable Daniel Webster, Secretary of State: with a Description of the Post-mortem Appearances, &c.* By John Jeffries, M. D *—Mr. Webster was of a sanguineo-bilious temperament, of a swarthy complexion, with straight black hair, with a large, athletic, and well-proportioned frame. He was five feet ten inches in height, and when in health weighed one hundred and ninety pounds. His appearance was peculiarly imposing, and the expression of his features, more particularly of his eye, was, perhaps, more powerful than that of any other man. He was nearly seventy-one years of age at the time of his death.

Mr. Webster, although endowed with an iron constitution, had been subject *for the past eighteen or twenty years to an habitual diarrhœa*, which, commencing as an occasional looseness, had gradually increased upon him until

---

* The author is indebted to Dr. S. Parkman, for the arrangement of this paper from the notes read before the Suffolk District Medical Society.

for the last three years it was persistent; for this, he was accustomed, latterly, to use opiates generally in the form of a "cholera medicine," which appeared to be composed of sulphate of morphia and the compound spirits of sulphuric ether.

For about the same number of years he had been annually subject to a somewhat severe form of catarrh, commencing from the 6th to the 16th of August, and continuing until about the 1st of October. The only exception to the occurrence of th s was in 1839, when he was in Europe. He was sometimes confined by this for a short period, but usually continued his exercise and duties abroad. In the early years of this complaint, he did but little for it; but, latterly, he had adopted energetic treatment under medical advice, in the hope of preventing the annual visitation.

I n August, 1851, while at Franklin, N. H , whither he had gone for retirement, hoping, by a change of climate to escape his annual catarrh, he was attacked, after exposure to the damp ground, with gout in his feet, mostly in the great toes, he was so far relieved of this, however, as to take a journey to the White Mountains; but, on his return to Franklin, the gout returned in a more severe and general form.

On the 9th of September, he came to Boston and placed himself under the writer's care. At this time, his complexion was sallow, and he had lost considerable flesh; his eyes were red, and his countenance indicative of great uneasiness; his pulse was full, quick, and firm; his nights were distressing and restless; there was constant thirst; the bowels were irritable, and, although without appetite, he was taking food without restraint, and, by advice, using stimulating drinks freely. He was also taking iodide of iron with hydriodate of potass. and minute doses of oxide of arsenic as a preventive of the catarrh. He had also used some remedies for the gout, and frequently resorted to opiates for his diarrhœa. With some difficulty he was induced to relinquish all these medicines, to restrict himself to the simplest food, and to retire to Marshfield for recreation and exercise. In September, he returned to Washington, expressing himself as being "perfectly well," having implicitly followed the directions given. During the winter of 1851 and 1852, he transacted a vast amount of business at the seat of government; being, however, frequently under medical treatment for his diarrhœa. He failed in flesh and strength towards the spring; and, in the latter part of April, went to Marshfield in hopes of recruiting.

On the 6th of May, 1852, he was thrown from his wagon, falling forward upon his hands, and striking his head wi h much force upon the ground. He was for some time insensible, but soon recovered perfect consciousness. On the 20th he came to Boston, and was visited by Dr. J. Mason Warren in consultation. He was found to have injured the joints of both wrists, the left more severely, without any apparent displacement or fracture; there was considerable swelling and great ecchymosis of the whole forearm, with frequent severe paroxysms of pain through the joint; there was also a slight flesh wound near the right temple. He made no complaint of uneasiness in the head. On the 24th May, he addressd his fellow citizens in Faneuil Hall, being then suffering under great general debility. In July at the time of his public reception at Boston, he was suffering more than usual from his diarrhea, and was under medical treatment to enable him to go through the fatigues of that occasion.

On his return from Washington to Marshfield, in September, he took cold in Baltimore, and first complained of the sypmtoms connected with his final illness. On the 20th of September, he drove from Marshfield to Boston, a distance of thirty miles for Medical advice.

It was then observed that he had lost much flesh, which gave to his large eye a somewhat unnatural prominence. His face was pale with a peculiar sallowness; but there was no jaundice at this or any other time. He rose from the recumbent posture slowly and with some apparent difficulty, and he had the aspect of a very sick man. He stated that he had been more than usually unwell for a week or more; he complained of uneasiness on the left side of the abdomen, with consequent difficulty of lying on that side; there was also sometimes a sense of tightness across the lower part of the abdomen. The bowels were still loose, but not quite so irritable; the appetite was wholly gone; the skin was commonly very dry, and there was a constant dryness of the tongue and fauces, with much thirst. The tongue was covered with a light brown coat; the pulse was 106, quite full, but easily compressed, somewhat jer ing, with four intermissions in a minute. The urine was scanty, high-coloured, and very turbid after standing, not coagulating by heat. The abdomen was much distended and resonant from flatus at almost every part, but particularly at the arch of the colon; there was flatness in the hypograstric and iliac regions, and signs of dropsical effusion were thought to be perceived. The edge of the liver, more distinctly felt than at any subsequent period, was firmer than natural, but without tenderness on pressure. Neither was there soreness at any part of the abdomen. The feet and legs were œdematous, considerably so about the ankles. There was some soreness of the soles, especially under the ball of the great toe. There was a similar soreness in the left thumb and wrist which had been most injured by the accident. He had also flying pains about his lower limbs and body, described as similar to those previously experienced from the gout. The usual course of action of the bowels was a dejection at five or six P. M.; another at nine, and a third at from two to four A. M.; these, especially the last, were urgent, loose, and with much flatus. After the morning dejection, he took a portion of his usual "cholera medicine," which gave him relief.

He returned to Marshfield the next day, the 21st, with the following directions: To abstain from all mental labour, and to avoid fatigue in bodily exercise. The diet to be tea with bread and butter, morning and evening, and a little animal food at dinner, with one vegetable.

To take two drachms of castor-oil, and an equal quantity of lemon-juice, every second or third day, if troubled by distension, or if the bowels did not act kindly. To take a cardiac mixture twice daily, and a pill of one grain of acetous extract of colchicum with two grains of camphor each night.

To have the abdomen gently rubbed, and a hot alkaline bath applied night and morning; the feet and legs, after being smeared with olive oil, to be rubbed with warm spirit twice daily.

On the 28th, 2[illegible]th, and 30th, he was visited at Marshfield, and was found with much the same symptoms, except that the abdomen was more tense and flat, and heret was well-marked fluctuation, with some soreness of the left side, for which five leeches were applied with relief. The urine was a little less scanty and turbid. Hehad continued to come down stairs, and one day had driven for four hours with visitors; but this had increased the dieffiulty of the bowels, and much fatigued him. He had had a little headache in the latter part of each afternoon; and he also spoke of a feeling of sinking and exhaustation, which came on about two o'clock each day.

On leaving him on the 30th, he was advised to substitute one-sixteenth of a grain of morphia for the "cholera medicine;" to have the abdomen embrocated with spirits of turpentine, diluted with common spirit; to take a pill of four grs. compound extract of colocynth, if the bowels required more action; to have eight or ten leeches applied to the right hypochondrium, if the bowels were more uneasy, and to take two teaspoonsful of brandy, with water, at 2 P. M., each day, if he felt exhausted

During the writer's absence, he was attended by Dr. John Porter of Marshfield, from whom frequent reports of his condition were received.

On the 6th of October, he was visited in consultation with Dr. James Jackson, of Boston. The symptoms continued much the same. Mr. Webster was about the house, though he had not been out. The opinion was expressed and concurred in, that there was ascites, dependent upon grave disease of some abdominal organ, which would ultimately prove fatal; although some relief might be obtained.

It was decided to substitute a mild tonic for the cardic mixture; to give one grain of squills night and morning, to be increased if the stomach could bear it; to continue the morphia; and to double the amount of brandy; encouraging him also to take a little animal food.

The symptoms continued much the same until the morning of the 11th, when, on coming down stairs for a drive, he became faint, with nausea and retching, vomiting a little mucus. Visited at seven P. M., he complained of extreme distress at the præcordia, for which he was urgent to have relief; the nausea had subsided. A teaspoonful of castor-oil, with one-sixteenth of a grain of morphia, was directed by which the pain was relieved, and an evacuation obtained about 2 A. M., consisting of much fecal matter, with very dark bile and gelatinous mucus. All medicines but morphia were omitted; castor-oil being directed to be used if the præcordial distress should return.

An annoying symptom, consisting of pains about the feet, of which he had previously complained, was noticed to increase in severity from this time. He continued tolerably comfortable, and able to come down stairs every day; and sometimes to transact considerable business. He was feeble and emaciated, but his spirits were buoyant. Throughout his sickness it was noticed that he did not bend his body forward in rising, but was raised with the body erect; and more than once upon being assisted to walk, he had said that he felt as if he should fall forwards.

On the 19th, there was a manifest falling off; he had several copious dejections, which were thought to contain some blood, and he had lost also two turns of retching; by these he was much enfeebled.

On the 21st at 5 A. M., the dejection consisted of a large quantity of fecal matter, with much bilious and bloody fluid. At 7 A. M. he had another similar dejection, with bilious vomiting. Nausea and retching now became prominent symptoms, and he became more and more feeble, until at 5 P. M., on the 22d, he vomited about a pint of fluid blood with some coagula. During the night the vomiting became more urgent, always with blood; and at 2½ A. M. he had a copious ejection of fluid blood. By all this he was much exhausted. The vomiting of blood continued very profusely; and whenever he attempted to speak, he was interrupted by hiccough or retching.

On the morning of the 23d he announced himself conscious of his situation, and said, "I shall die to-night." From 9 until 1½ P. M. he remained free from vomiting. He was at this time visited by Dr. James Jackson, who had frequently been consulted during the progress of the disease. The vomiting of blood recurred during the afternoon. Dr. J. Mason Warren arrived towards night, and remained until the patient's death. Mr. Webster continued thus gradually sinking from the loss of blood by vomiting, retaining the power of utterance, until midnight, and some evidence of consciousness until 1, A. M., and sinking gradually, without convulsion, cold sweat, or haze of the eye, expired at thirty-five minutes past two, on the morning of Sunday, October 24.

For the last two days he was supported by such stimulants as he could bear, and was quieted by opiates when required.

The autopsy was made by Dr. J. B. S. Jackson, who furnishes the following report:

*Autopsy* thirty-two hours after death; present Drs. Jeffries, Porter, J. Mason Warren, Wyman, Parkman, and Jackson.

The emaciation was very marked, as shown by the state of the integuments and muscles; the latter being wasted, pale and flabby.

*Abdomen.*—The peritoneal cavity contained eleven pints of serum. There were also old and strong adhesions about the spleen, the gall-bladder, the cæcum, and to a small extent between the left extremities of the arch of the colon and the parietes of the abdomen.

The stomach was distended, and contained half a pint of very dark blood, abont one half of which was in a state of soft coagulum; and this was the only appearance that was found of coagulum in any part of the body. The mucous membrane was deeply stained by the contents, generally rather soft, and in the pyloric portion somewhat mamellonated. The intestines were opened throughout, washed, and fully examined with reference to the diarrhæa that had so long existed. Blood was found throughout in very considerable quantity as far as tne descending colon, below which there was no trace of it; in the large intestine it was altered as usual in color. Mucous membrane stained by the contents so far as blood extended. In the large intestine were numerous herniæ of the mucous membrane, so common in this situation; from many of these small masses of feces or of mucus could be forced out, and these were the only traces of feces that were found. Otherwise, the mucous membrane of the intestines appeared quite healthy; there being nowhere any ulceration to explain the diarrhœa, nor ecchymosis connected with the hemorrhage.

The liver was, throughout, very markedly granulated; dense, and contracted in size; the color externally was greenish or bronzed, but internally everywhere of a pale red; showing, as we may not very unfrequently observe, the inappropriateness of the term "cirrhosis," which would generally have been applied to the present case. Weight of the organ, three pounds and one-third, avoirdupois. Bile, in the gall-bladder, and of a tarry consistence.

Spleen small, pale and shrivelled. Investing membrane to some extent opaque, white, thickened, and condensed; this change being probably due to the old peritoneal affection.

Kidneys and pelvic organs healthy.

*Thorax.*—Old pleural adhesions over nearly the whole of the right side; none on the left. Lower lobe of the left lung and the two lower lobes of the right much congested, and very dark; a change that undoubtedly occurred toward the close of life, being simply passive.

Heart flaccid; very little blood in cavities, and this was quite liquid. Slight disease of aortal valves, but organ otherwise healthy. Foramen ovale; a small valvular opening existed. Aorta not ossified, except to a small extent in the abdomen.

*Head.*—The membranes of the brain were most remarkably diseased. In the cavity of the arachnoid was a layer of fibrine which covered almost entirely and about equally the convexity of both hemispheres; it did not extend, however, beneath nor between them, nor about the cerebellum. In the recent state, it had a rather dull, yellowish, infiltrated, œdematous appearance; being one-fourth of an inch in thickness over the upper surface, but becoming gradually more thin on the sides, where it terminated in a thin edge. The adhesion to the dura mater was in some parts quite close; but it was generally very readily stripped off, and left the arachnoid with its usual polish. It was more adherent to the subjacent membrane; this last being irregular, and having generally a clouded and slightly opaque appearance, with many milk-white spots, but without any appreciable thickening. The quantity of serous effusion into the membranes was altogether large. The subarachnoid tissue corresponding to the layer of fibrine above described was infiltrated with a straw colored serum in some places, separating the convolutions from each other; this separation was quite remarkable at the posterior part of the right cerebral hemisphere, on its upper surface and near the meridian line, there being also a slight depression at this part. The dura mater adhered firmly to the calvaria, but was healthy in structure, as were the membranes otherwise; there was, however, a serous infiltration into each plexus choroides; though no more, if not less, than usual into

7

the lateral ventricles. No appearance of recent meningitis; and no effused blood or cysts in or about the false membrane. The brain itself was perfectly healthy; and the arteries at the base very nearly so. Cranium healthy. Over the right frontal region a scar existed, the result of the injury that occurred last May; integuments not otherwise remarkable.

A portion of the fibrine from the arachnoid cavity having been removed for microscopical examination, it was found, some hours afterwards, and when the serum with which it had been infiltrated was absorbed, to have almost the consistence of one of the natural tissues of the body; being strong enough to bear considerable traction; it also appeared then to have somewhat of a laminated structure, and bloodvessels were distinctly seen in it even with the naked eye. Dr. Wyman found it "organized, and, in some places vascular. Under the microscope, the lymph was resolved into minute fibres, like those forming the white fibrous element of areolar tissue, and including in their meshes large numbers of minute granules."

Recapitulating the points of interest in this case, it will be observed that the immediate cause of death was hemorrhage from the stomach and bowels. For this, no source could be found in the lesion of any vessel; it must therefore be regarded as a simple exhalation dependent upon a disorganization of this fluid, indicated, moreover, by the almost entire absence of coagulation. The relation of this hemorrhage to the disease of the liver will also be noted as coinciding with previous experience; it being well known that, in certain cases where there is an altered action of this organ, there is a tendency to disorganization of the blood, manifesting itself thus in hemorrhage.

The morbid appearances observed in the cerebral membranes possess, also, very great interest in several aspects. It will be unnecessary to dwell upon the particular appearances carefully described above. A very full and clear description of these interesting forms of extravasation has been published by Mr. Prescott Hewitt, in the twenty-eighth vol. *Medico-Chirurgical Transactions of London*, and the appearances, in this case, coincide with those there described. Grisolle (*Pathologie Interne*, vol. i.) has also well described this affection, after the original descriptions of Serres, Baillarger, Boudet, and Prus, who were the first to call attention to this particular lesion The case of Mr. Webster may be regarded as unique˙ however, in this respect, that no impairment of the power of the nervous system was observed before death; for although a few symptoms, such as his mode of locomotion, his sense of falling, and a slight hesitation of his speech, may now be remembered and connected with this condition, it will be sufficient to prove the entire absence of any suspicions of the kind during life, to state that the brain would not have been examined at the autopsy, except for the desire of making the measurements, &c., recorded below. The connection of this meningeal hemorrhage with the cirrhus of the liver will also give rise to interesting speculation; for although it is quite probable that the origin of the effusion should be ascribed to the accident in May, still, it is not unlikely to be remotely dependent upon the disorganization of the blood consequent upon the disease of the liver, since among Mr. Hewitt's cases there are some recorded where an effusion quite equal to this took place in connection with a cirrhus without any injury at all. It is possible, moreover, that the accident may not have been the cause of the effusion, which may have taken place since that time; but, in the presence of what would appear adequate cause, it will be unnecessary to look beyond.

In the treatment of the disease, attention was particularly directed to the duodenal obstruction, relief from which was obtained by the laxatives occasionally administered, and these, with opiates, were almost the only important medical agents.

The following very interesting account of the cranial cavity and brain is furnished by Dr Jeffries Wyman:

The dimensions of the brain, as indicated by the measurements of the cranial cavity,* were as follows:

| | | | | |
|---|---|---|---|---|
| Longitudinal diameter, | : | : | $7\frac{7}{8}$ | inches. |
| Transverse " | : | : | $5\frac{3}{4}$ | " |
| Vertical " | : | : | $5\frac{1}{2}$ | " |
| Breadth of occipital fossa, | : | : | $4\frac{3}{4}$ | " |
| " frontal " | : | : | 5 | " |

* In consequence of its flaccidity, the natural diameters of cerebral substance are no longer preserved after the brain is removed from the cranial cavity; its diameters are, therefore, more correctly measured by determining those of the cavity which it filled.

The posterior clinoid processes were seven-eights of an inch in front of the centre of the cranial cavity.

The circumference of the head was 23¾ inches, and the distance from the meatus of one ear to that of the other, over the top of the head, was 15 inches.

The capacity of the cranium, determined according to the method adopted by the late Dr. S. G. Morton, of Philadelphia, was 122 (one hundred and twenty-two) cubic inches.

The substance of the brain was firm to the touch, and, as regards color and consistence, appeared to be healthy. The depth of the spaces between the convolutions was, on the vertex seven-eights of an inch, and the "cortical" or gray substance was three-sixteenths of an inch in thickness.

The corpus callosum, or the great cerebral commissure, was large, measured four inches in length from before backwards, and at the central portion was one-fourth of an inch in thickness.

The pineal body, as in the great majority of instances, contained calcareous concretions.

The weight of the brain, including the cerebrum, cerebellum, and medulla oblongata, as far as the lower extremity of the pyramids was (in avoirdupois):

| | Lbs. | Oz. | Drachms. | Grs. | Grains. |
|---|---|---|---|---|---|
| Brain (encephalon)* | 3 | 5 | 8 | 17.75= | 23,424.0 |
| Cerebrum : : | 2 | 14 | 7 | 14.09= | 20,330.5 |

The measurements which have been given above, are almost without exception of unusual proportions. The average length of the cranial cavity does not exceed six and a half inches; its transverse diameter is five inches, and the vertical a little less.†

The cranial capacity was very unusual, the largest which has yet been recorded, though measurements in

* In Troy weight the result was as follows:

| | Pounds. | Ounces. | Pennyweights |
|---|---|---|---|
| Brain, : : : : : | 4 | 0 | 16 |
| Cerebrum, : : : : : | 3 | 6 | 6 |

† Dr. Morton gives the average diameters for European and Anglo-American skulls as follows: Longitudinal, 6½; transverse, 5½; and vertical, 5 inches. These measurements, however, are external, and include the thickness of the scull and would, therefore, be too large, by cubic inches have, as yet, been made by comparatively few observers. In Dr. Morton's Tables of the measurements of 623 crania of different nations, including Caucasians, Mongolians, Malays, Americans, and Negroes, only four instances occur in which the capacity exceeded one hundred cubic inches; of these, the largest were one English scull, measuring 105, and one German 114 cubic inches. According to Dr. Morton, the average capacity for the Teutonic family (including English, Germans, and Anglo-Americans) is 92 inches.*

The two superficial measurements of the head were very nearly those of Cuvier, the circumference of whose head was 22 inches 4 lines (French), and the measurement from ear to ear over the top was 15 inches. The circumference of Napoleon's head is reported to have been 23 inches.

The weight of the brain deviated much less from the average than the measurements; it was entirely out of proportion to the unusual dimensions of the cranial cavity. The average weight of an adult healthy male brain is 49½ ounces, or 3 pounds 1½ ounces avoirdupois.† As has been already stated, there existed an effusion of serum into the subarachnoid areolar tissue, and of serum and lymph into

---

the thickness of the cranial walls, to represent the size of the brain. *Human Anatomy*, p. 70: Philadelphia, 1849.

Cruveilhier, following Bichat, makes them somewhat less than those given in the text; his mode of measurement, however, does not give the greatest dimensions of the cranial cavity. See his *Traite d' Anat. de l'Homme*, t. i. p. 140. Paris, 1843.

* Catalogue of Skulls of Man and the Inferior Animals in the collection of Samuel George Morton, M. D., Philadelphia, 1849. See Comparative Table on page viii., and Specimen No. 434.

Dr. J. B. S. Jackson, in the Descriptive Catalogue of the Anatomical Museum of the Boston Society for Medical Improvement, has given the measurements of thirty sculls of different nations, the largest of which, a Theban and a Negro, were 95 inches each. Of ten Hindoo skulls, measured by Dr. S. Kneeland, Jr., the largest, that of a Rajah, contains 92 inches. Proceedings of Boston Soc. Nat. Hist., vol. iii., p. 213.

† This is the result of observations on two hundred and seventy-eigh adult healthy male brains. See Sharpey's Quain's Anatomy, Dr. Leidy's edition, vol. ii. p. 186. This determination is based on the combined observations of Reid, Sims, Tiedemann, and Clendinning, which are all reduced to avoirdupois weights.

the arachnoid cavity. The lymph had existed a long time, it covered the convex surface of the cerebral lobes, was a quarter of an inch in its thickest portion, and extended to the sides, where it became quite thin. Both serum and lymph, there can be no doubt, encroached upon and occupied the space once filled with cerebral substance. The weight given above, therefore, cannot be regarded as being equal to the weight of the brain in a state of health. This last we now have no means of determining except by an approximation, which has been made in the following manner, in accordance with a suggestion by Professor Treadwell, of Cambridge:

The specific gravity of the brain is, according to Cruveilhier and others, 1030, water being 1000. A cubic inch of water weighs 252.5 grains, and 122 cubic inches (the cranial capacity), would equal 30,805 grains, to which must be added 3 per cent., or 924 grains (the excess of specific weight of brain over water), which gives 31,829 grains as the full capacity of the cranial cavity in weight for cerebral substance. The brain, however does not actually fill the whole cavity; a correction must, therefore, be made for the spaces occupied by the tentorium, falx, sinuses, the dura mater of the calvaria, and the cephalo-spinal fluid at the base of the brain. If we deduct eight ounces for such spaces, we shall have an actual weight of 28,329 grains; or, if nine ounces are deducted, 27,891 grains. Taking the last approximation as the one the least liable to error of excess, Mr. Webster's brain will be found to rank among those whose brains are generally cited as instances of remarkable size, as follows:

| | Lbs. | Ozs. | Drs. | Grs. | | Grs. | | Ozs. |
|---|---|---|---|---|---|---|---|---|
| Cuvier* | 4 | 0 | 5 | 10 | = | 28,147 | = | $64\frac{1}{3}$ |
| Webster | 3 | 15 | 12 | 0 | = | 27,891 | = | $63\frac{3}{4}$ |

* In the official report of Cuvier's *post-mortem* examination, the weight of the brain is given as 3 livres, 11 ounces, 4.5 gros, poids de marc, or old French weight; this, reduced to avoirdupois, gives the weight in the above table, It has, however, been differently stated by physiological writers. Tiedemann reports it as 3 lbs., 11 ozs., 4 drs., 40 grs., avoirdupois. (Memoir on the Brain of the Negro., Philos. Trans. 1836, p. 502.) This erroneous computation has been often repeated; and is the one given in the Cyclop. Anat. and Physiology, Art. Nervous System, p. 664, and in other works. It is correctly stated in Sharpey's Quain's Anatomy.

| | Lbs. | Ozs. | Drs. | Grs. | | Grs. | | Ozs. |
|---|---|---|---|---|---|---|---|---|
| Abercrombie* | 3 | 15 | 0 | 0 | = | 27,562 | = | 63 |
| Spurzheim† | 3 | 7 | 1 | 0 | = | 24,089 | = | $55\frac{1}{16}$ |
| Dupuytren‡ | 3 | 1 | 10 | 27 | = | 21,738 | = | $49\frac{11}{16}$ |

The brains, the weights of which (in avoirdupois) are included in this table, are not the only ones on record remarkable for size. In the table of Dr. Sharpey, already quoted, there are enumerated as weighing between 55 and 59 ounces, avoirdupois, inclusive, 28 brains; and between 60 and 65 ounces, 7.§ Nothing is said of the individuals from whom they were taken; of the two largest, one weighed 63 and the other 65 ounces; it is not improbable that these were the brains of Abercrombie and Cuvier; 63 ounces being precisely the weight of the former. In making out the table, all instances with fractional parts were classed with the next integral number; and, as Cuvier's brain weighed over 64 ounces, it would rank as 65 ounces. If this be not the explanation, then there is on record a larger healthy brain than that of Cuvier.

---

* Quoted from Sharpey's Quain's Anat., vol. ii., p. 187.

† Anatomical Report on the Skull of Spurzheim, read April 2, 1835, before the Phrenological Society of Boston, by N. B. Shurtleff, M. D. Annals of Phrenology, vol. ii., p. 72: Boston, 1835.

‡ Dupuytren's brain was really not remarkable for size, being only two drachms above average; it is generally erroneously reported at 4 lbs. 10 ozs. Troy. An official report, signed by Broussais, Cruveilhier, Husson, and Bouillaud, which may be found in *Revue Medicale*, 1835, states it to have weighed only 2 livres, 14 ounces, poids de marc. This, reduced to avoirdupois, is equal to the amount given in the table.

§ In estimating brains by weight, it must be borne in mind that quantity, and not quality, is considered; the anatomist has no means of determining quality. The head of Byron may be cited as an instance where small size was associated with great activity. Lord Napier informs us, that of fourteen persons who dined with him on one occasion, not one could wear Byron's hat. Napier's servant, who had the smallest head in the 90th Regiment, so small that he required to have his caps made expressly for him, tried on Byron's hat, and found that it just fitted him. See Moore's Life of Byron. In Dr. Bruno's report of the autopsy of Byron, his brain is said to have weighed "six pounds (*mediche*)." See Count Gamba's Narrative of Byron's last Journey to Greece, p. 271, London, 1825. This must be an error, if the pounds are those of apothecaries' weight. The above anecdote shows that his head was not large. Thorwalsden's bust does not give it unusual elevation; and Moore states that it was "disproportionably small." His habit of shaving off his hair gave it an appearance of elevation.

